# The New Hospital for Women

**Published:** 1916-12-16

**Authors:** Alfred Keogh


### THE NEW HOSPITAL FOR WOMEN.

### Sir Alfred Keogh, K.C.B., on its Jubilee and Value.

It is a good thing, even at a time like the present, when most of us—all of us, perhaps—are engaged in work of another kind, that we should assemble on an occasion like this to rejoice with the governors and the staff of this hospital on the attainment of their jubilee anniversary. I am old enough to remember the difficulties which women met with when they first ventured on the career of medicine. There are very few people in this room who will have taken part in those very early days when women were practically—and later on were actually—forbidden entry into a medical school in this country. It is a great thing, I think, that we should come to this hospital—which is called "New" in much the same way as New College, Oxford, is so called—and realise that this is the first, the oldest, institution in this country which had women doctors upon its staff. It is not only that the hospital itself may be proud of that, and ought to be proud of it, but all women, not merely in our own country, but all women throughout the world, should rejoice with a hospital of this kind upon the attainment of its jubilee. It marks, practically, the beginning of the admission of women to the hospital staffs in this country, a beginning which, till now, has not made very much progress.

Now, out of a great war like this, great good must come, and amongst the many things which will come out of it, I venture to think that the definite position of women in public affairs of this country will be one of the most notable. I, personally, ladies and gentlemen, have every reason to be proud of the work which medical women did before the war. I remember, some little time ago, when it was proposed to me, or, rather, perhaps I should say I proposed it, that a large hospital in London should be staffed by women doctors, there was nothing to me very extraordinary in that: women physicians and women surgeons are, as far as I know, quite as good as men physicians and men surgeons. But, to my amazement, I found the spirit of fifty years ago almost as rampant as it was in those days. There was a good deal of opposition to this idea. I hardly dared tell the staff of that hospital—and I have never told them—(laughter)—how great were my difficulties in seeing my way through on that occasion. All sorts of impossible things were suggested to me, but, thank Heaven, we carried through, and the hospital to-day in London is one of which the country may really very well be proud.

So much for the women's movement in this country. And now for the hospital. I confess that I never hear of the establishment of a hospital, or a proposal to extend it, without a certain regret. As a matter of fact, hospitals represent a certain failure in science. The curative side of medicine has, of course, been the side which has been developed, and to which attention has been paid, for long, long ages. Preventive medicine is new. Preventive medicine in this country may be said to have begun after the Crimean War. And I mention that specially because it is an interesting fact that the development of sanitation in this country, and the further development which we shall expect after this war, will have been begun and continued in consequence of two great wars. The history of preventive medicine in this country is the history of preventive medicine in the Army. I do not want to go into these things, ladies and gentlemen, because my time is limited: I am only allowed a few minutes to say anything to you. It was in the Crimean War when the celebrated Barracks Hospital Commission of Lord Herbert was established, that public health got an impetus in this country, which has retained ever since.

### The Results of Science Failure.

When I say that the establishment and extension of hospitals in this country represents, in a certain sense, the failure of science, what I mean to say is, that if preventive medicine in this country were developed in the way it ought to be, it is quite possible—as a great practical fact, not merely as a question of theory—that many of our hospitals in this country might remain empty. I have not seen the statistics of this institution, but let me take, for instance, one particular disease, which has defied all efforts. I have no doubt that the physicians and surgeons inside this hospital are thoroughly well acquainted with the ravages of such a disease as cancer. Now, cancer slays myriads of people, and of cancer we know nothing, or practically nothing. In my own opinion—and I speak very humbly in the presence of a great pathologist—the investigation of cancer has passed too much into the hands of pathologists, and not sufficiently into the hands of physiologists. Now, women, of all people, ought to be particularly interested in this disease. The physicians and surgeons of this hospital must have an unfortunate experience of it, and I, for one, earnestly hope that, as the result of the organisation of scientific research which has been essential for the conduct of this war, not only in the direction of medicine, but in all directions, that the organisation of research will be continued after the war, as it was practically begun after the Crimean War. But, ladies and gentlemen, that is a Utopian idea. This hospital must continue, it must be enlarged, and it ought to be helped. There is an interesting point about this hospital which makes it unlike any other hospital I know of, and that is, that it has an annexe in the country. All of us know, of course, that the ideal thing is to have all hospitals in the country: but that is an ideal impossible of attainment in a great many instances. But this hospital has attained it, and endeavours to remove its operation cases as quickly as possible out of this building and send them into the country. Obviously the ideal itself was restricted from want of funds. Others, no doubt, will tell you in detail what is required. But I do think that, on an occasion like this, when we are celebrating the jubilee of what is virtually the emancipation of women in this country, and when we are commemorating the existence of a hospital which has done so much for women in this great city, and continues to do it, and that it has a staff which is representative of all that is best in the medical profession on the women's side, I do think it is worthy of your support.

I have come away to-day for a few moments—I very seldom come out of the War Office—because I could not stay in there this afternoon when I heard that this New Hospital for Women, representing, as it does, women's work, and not only the work of medical women, required support from the public, and I was asked if I would say a word in connection with it. I have kept you rather longer than my time permitted, but I do feel very keen on this subject, and I earnestly hope that help will be forthcoming for this hospital, and that the appeal will not be made in vain.

